# Conversion of Open Unclassical Bariatric Metabolic Surgery into Laparoscopic Roux-en-Y Gastric Bypass: a Multimedia Article

**DOI:** 10.1007/s11695-023-06724-x

**Published:** 2023-07-08

**Authors:** Mohamed Hany, Mohamed Ibrahim, Anwar Ashraf Abouelnasr, Bart Torensma

**Affiliations:** 1 Department of Surgery, Medical Research Institute, Alexandria University, 165 Horreya Avenue, Hadara, Alexandria, 21561 Egypt; 2 Madina Women’s Hospital, Alexandria, Egypt; 3 Leiden University Medical Center (LUMC), Leiden, Netherlands

**Keywords:** Distalization, RYGB, Revision Surgery, Scopinaro’s Surgery, Informed Consent

## Introduction

Over time, bariatric metabolic surgery (BMS) has gone through significant changes. Procedures like ileocolic and jejunoileal bypass, vertical banded gastroplasty (VBG), and nonadjustable gastric banding have been abandoned due to complications and poor outcomes. Roux-en-Y gastric bypass (RYGB), sleeve gastrectomy (SG), adjustable gastric banding, and biliopancreatic diversion with duodenal switch replaced this procedure with known published results [1]. This report details a rare case involving an unconventional open procedure for weight loss, highlighting the revised approach and actions undertaken during the surgical intervention.

## Presentation and Pre-workup

A 38-year-old female complained of insufficient weight loss and regain after the previous open surgery in 2010 (BMI 58.1 kg/m^2^) presented at the Madina Women’s Hospital, Alexandria, Egypt. Although the patient presumed her surgery might be vertical banded gastroplasty (VBG), no operative data were accessible at the time of evaluation as the patient had previously been operated on in another center. The current weight was 150 kg (BMI 54.4 kg/m^2^). The current associated medical problems were hypertension, hyperlipidemia, and osteoarthritis. The patient lost − 20 kg in her first year to reach a BMI of 50.8 kg/m^2^. Food tolerance was reported to be good and even got better over time. After analyzing the patient’s history, specifically her bowel habits and stool consistency, it was determined that her surgery did not suggest a distal bypass procedure.

Following a multidisciplinary team consultation (MDT) involving a surgeon, nutritionist, and psychiatrist, as well as preoperative workup including imaging and endoscopy, the following findings were identified: (1) gastrojejunostomy and fundus and most of the stomach were present; (2) metal wire was observed on the lesser curve;( 3) the endoscope failed to reach the antrum; and (4) enteroenteric anastomosis was noticed by CT with a sort of roux diversion. Based on these findings, the patient provided informed consent for a laparoscopic exploration and authorized the surgeon to proceed with the best course of action, as determined by their professional judgment.

## Revision Surgery

During laparoscopic surgery, extensive adhesiolysis was performed, and a retro-colic gastrojejunostomy was found with the aid of upper GI endoscopy. The stomach was divided by a wire apparent during dissection, and they were not communicating.

Enteroenteric anastomosis was found during dissection but anastomosed earlier to the alimentary limb. Dissection of adhesions between bowel loops was performed. By counts of 5 cm, the whole bowel was counted towards a total common channel (CC) of 500 cm, alimentary limb (AL) of 110 cm, and biliary limb (BL) of 50 cm. In summary, the surgical team arrived at a diagnosis of an unclassical procedure (open RYGB with undivided pouch), whereby it presented with a large pouch, and upon exploration, a long common channel was discovered. Consequently, the surgical procedure revealed minimal gastric reduction and only a slight intestinal bypass. Our options were to reduce pouch and redo bowel lengths, the AL at the jejunojejunostomy was divided and transposed 200 cm distally to create a total alimentary limb length (TALL) of 405 cm, ensuring a CC of 300 cm, and BL became 250 cm. A drain was inserted. The anastomosis was tested, and mesenteric defects and colon mesenteric space admitting the alimentary limb were closed continuously with a barbed V-Loc™ PBT non-absorbable 2–0 suture (Medtronic, Mansfield, MA). All other stable lines were reinforced with Stratafix 3–0 suture (Ethicon, Johnson & Johnson, New Brunswick, NJ, USA). So, we converted this unclassical type of surgery to roux-en y gastric bypass (RYGB) with the long biliary limb.

### Postoperatively and Follow-up

The patient was hemodynamically stable, tolerating oral fluids, and was discharged on postoperative day two. The patient lost − 57 kg in the first year (BMI 33.8 kg/m^2^), and no bowel symptoms were noted, but frequent episodes of dumping without discomfort were present. The patient had normal laboratory values and still receiving multivitamins, iron, and calcium supplements.

## Discussion

The existence of uncommon surgical procedures, often characterized by their intricacy and the challenges associated with their comprehension, underscores the formidable obstacles bariatric metabolic surgeons encounter in their clinical practice. The success of any surgical procedure heavily relies on the implementation of well-established surgical techniques and guidelines. Devising uncommon surgical methods to suit a particular patient’s condition may lead to undesirable outcomes and significantly threaten the patient’s well-being. Therefore, bariatric metabolic surgeons must approach each case with the appropriate mindset and expertise, prioritize using evidence-based surgical techniques, and resist the temptation to do something unconventional again. This will minimize the risks associated with surgical procedures, ultimately, enhance the quality of care provided to patients with a BMS. This multi-media article highlights the importance of bariatric metabolic surgeons focusing on practicing known types of surgeries and adhering to the principles of bariatric metabolic surgery.

Our case was unclassical, and it was difficult to identify the type of procedure due to the preservation of the stomach with a wire in situ and incorrect limb lengths, including a CC of 500 cm instead of 50 cm known in biliopancreatic diversion (BPD) surgery. Furthermore, during exploration, it was also not considered a classical RYGB; MacLean et al. recommended a gastric pouch of 30 ml to achieve long-term weight loss, surgeons challenged the creation of a virtual pouch, and it must be isolated [2].

Our options were to reduce the pouch and adjust the bowel lengths, which is what we ultimately did. However, there is a potential danger of too much restriction with a distal bypass. Alternatively, we could have converted to a very distal operation, such as Scopinaro’s BPD, but we were concerned about the potential adverse effects. We adhered to the principles of TALL and CC length to lower the risk of nutritional deficiencies and increase the probability of weight loss for the patient.

BPD is uncommon worldwide, representing less than 2% of all BMS procedures [3]. It is also rare in our country, Egypt. For BPD procedure, it has some drawbacks: it is irreversible, involves a distal gastric resection, creates a blind duodenal stump that does not allow endoscopic access to the biliopancreatic limb area postoperatively, and has a short CC of 50 cm with malnutrition as consequence [4].

### Distalization

We used an RYGB with distalization in this revisional case. The technique involved dividing the AL at the entero-enterostomy and moving it closer to the ileocecal valve to shorten the TALL, the sum of the CC and AL.

Distalization will make BL longer, which improves weight loss outcomes, prevents weight regain, and resolve associated medical problems [5, 6]. As a result, in our case, the TALL was 405 cm (with a CC of 300 cm), and the BL was 250 cm. We performed direct distalization instead of standard RYGB for several reasons: The patient had severe obesity with a BMI of 54.4 kg/m2 at the time of revision surgery, justifying the use of distal RYGB. Distalization was chosen to increase the chances of better BMI reduction and to avoid the risk of future re-operation for insufficient weight loss and adhesiolysis due to the primary case being an open BMS with multiple adhesions.

A systematic review (SR) from 2022 that included 21 studies on the best TALL and CC strategy found that shortening TALL could affect weight loss outcomes. However, the SR concluded that a TALL of less than 400 cm with a common channel of less than 200 cm should be avoided due to severe protein malnutrition, observed in 3.4% to 63.6% of patients who required limb lengthening. Notably, these studies in the SR included primary and revisional surgeries, different limb lengths, and various techniques for measuring limb length, contributing to their heterogeneity [7].

## Conclusion

When rare and unclassical cases are presented, it underscores the importance of an MDT and diagnostic preoperative workup to navigate challenging scenarios.

Safe distalization of RYGB requires avoiding limb lengths under 400 cm and common channels under 200 cm to prevent severe protein malnutrition and re-operations.

## Supplementary Information

Below is the link to the electronic supplementary material.

Supplementary file1 (MP4 269025 KB)
